# Decoy receptor 3 is involved in epidermal keratinocyte commitment to terminal differentiation via EGFR and PKC activation

**DOI:** 10.1038/s12276-022-00762-8

**Published:** 2022-04-27

**Authors:** Nan-Lin Wu, Duen-Yi Huang, Shie-Liang Hsieh, Yang-Shia Dai, Wan-Wan Lin

**Affiliations:** 1 Department of Dermatology, MacKay Memorial Hospital, Taipei, Taiwan, ROC; 2 Department of Medicine, MacKay Medical College, New Taipei City, Taiwan, ROC; 3 MacKay Junior College of Medicine, Nursing, and Management, Taipei, Taiwan, ROC; 4 Institute of Biomedical Sciences, Mackay Medical College, New Taipei, Taiwan, ROC; 5 Department of Pharmacology, College of Medicine, National Taiwan University, Taipei, Taiwan, ROC; 6 Genomic Research Center, Academia Sinica, Taipei, Taiwan, ROC; 7 Department of Dermatology, National Taiwan University Hospital, Taipei, Taiwan, ROC; 8 Graduate Institute of Medical Sciences, Taipei Medical University, Taipei, Taiwan, ROC; 9 Department of Pharmacology, National Defense Medical Center, Taipei, Taiwan, ROC

**Keywords:** Differentiation, Growth factor signalling

## Abstract

Decoy receptor 3 (DcR3) is a soluble receptor for Fas ligand, LIGHT and TL1A, but it also exerts effector functions. Previously, we found that DcR3 is upregulated in the serum and lesional skin of patients with psoriasis and is upregulated by EGFR activation in proliferating primary human epidermal keratinocytes. However, the functional role of intracellular DcR3 in keratinocyte differentiation is still incompletely defined. Herein, primary cultured human epidermal keratinocytes were differentiated by phorbol 12-myristate 13-acetate (PMA) treatment, calcium treatment and cell confluence, which are three standard in vitro differentiation models. We found that the constitutive expression of the DcR3 gene and protein was progressively suppressed during terminal differentiation of keratinocytes. These changes were correlated with downregulation of EGFR activation during keratinocyte differentiation. EGFR inhibition by gefitinib further decreased confluence-induced suppression of DcR3 mRNA expression, and, vice versa, knocking down DcR3 expression attenuated EGFR and EGFR ligand expression as well as EGFR activation. Under conditions without a change in cell growth, DcR3 silencing reduced the expression of involucrin and transglutaminase 1 but enhanced the induction of the terminal differentiation markers keratin 10 and loricrin. Of note, DcR3 interacted with PKCα and PKCδ and enhanced PKC activity. In keratinocytes with PKCα and PKCδ silencing, differentiation markers were differentially affected. In conclusion, DcR3 expression in keratinocytes is regulated by EGFR and forms a positive feedback loop to orchestrate constitutive EGFR and PKC activity. During differentiation, DcR3 is downregulated and involved in modulating the pattern of terminal differentiation.

## Dermatology: abnormal development of skin cells in psoriasis

A protein linked to cancer and various inflammatory diseases may also be an important driver for the skin condition in psoriasis. The outer surface of the skin is formed by cells called keratinocytes, which transition from a highly proliferative state to a fully mature state where they no longer divide. This developmental process is disrupted in psoriasis. Researchers led by Wan-Wan Lin at National Taiwan University, Taipei, have now identified a prominent role for a protein called decoy receptor 3 (DcR3), which is a biomarker for a variety of disorders and is also abnormally expressed in keratinocytes in psoriatic lesions. Lin and colleagues demonstrated that DcR3 interacts with multiple cellular signaling pathways that coordinate cell differentiation. These findings reveal how aberrant DcR3 activity might lead to the abnormal keratinocyte developmental behavior observed in psoriasis.

## Introduction

Decoy receptor 3 (DcR3) is a member of the tumor necrosis factor receptor (TNFR) superfamily and is a 33 kDa glycosylated soluble factor lacking a transmembrane domain. DcR3 is able to exert its function via its decoy functions, including neutralizing Fas-ligand (FasL), lymphotoxin-like; exhibits inducible expression; and competes with HSV glycoprotein D for HVEM, a receptor expressed by T lymphocytes (LIGHT), and TNF-like molecule 1 A (TL1A) to inhibit apoptosis and promote angiogenesis. DcR3 has been reported to be elevated in serum and tumor samples of different cancers and is regarded as a biomarker in cancer progression^1–3^. A high DcR3 serum level has been established as an index of poor prognosis for cancer patients. Recently, DcR3 has also been shown to perform various nondecoy (non-neutralizing) functions by acting as an effector molecule via interactions with heparan sulfate proteoglycans (HSPGs), such as syndecan 2 and CD44v3^4–6^. DcR3 has been suggested to be a critical factor involved in the pathogenesis of multiple autoimmune and inflammatory diseases in addition to cancers^3^. The serum level of DcR3 is regarded as a biomarker of inflammation status in various diseases, including Crohn’s disease^7^, systemic lupus erythematosus^8^, rheumatic diseases^9^, acute respiratory distress syndrome^10^ and kidney diseases^11^.

Epidermal keratinocytes are the principal cellular component in the skin; these cells progressively differentiate from proliferative cells in the stratum basale to form the outermost layer of the keratinized stratum corneum^12^. To accomplish intact and functional cornification, epidermal keratinocytes need to express distinct differentiation marker proteins, such as transglutaminase (TGase), involucrin, keratin 1/10, loricrin and profilaggrin^12,13^. The epidermal growth factor receptor (EGFR) expressed in keratinocytes plays a unique role in controlling skin cell proliferation, differentiation, disorders and cancers^14^. During keratinocyte differentiation, EGFR expression and activity are downregulated^15,16^. Aberrant activation of EGFR leads to overproliferation of keratinocytes, contributing to psoriasis, while EGFR inhibitors switch keratinocytes from a proliferative to a differentiative phenotype, thus affecting epidermal development and barrier function^17^.

Regarding skin biology, DcR3 is detected in primary human keratinocytes^18,19^, and clinical studies have shown that the DcR3 level is elevated in the serum of patients with psoriasis and that DcR3 is overexpressed in the epidermal layers of psoriatic skin lesions^20,21^. Moreover, we previously demonstrated that EGFR activation is involved in upregulating DcR3 gene expression in keratinocytes through the NF-κB pathway^21^. Although DcR3 is present in keratinocytes and is a skin marker for psoriasis, its biological action in keratinocytes remains elusive. Therefore, in this study, we explored the role and molecular mechanism of DcR3 in keratinocyte differentiation and its relationship to EGFR. Our data revealed that DcR3 can cooperate with EGFR to modulate keratinocyte differentiation; in addition, for the first time, we identified protein kinase C (PKC) isoforms as intracellular targets of DcR3 in the regulation of keratinocyte differentiation. The novel functions of DcR3 in the regulation of keratinocyte terminal differentiation may account for its pathological role in psoriasis.

## Material and methods

### Cell culture

Primary normal human epidermal keratinocytes (NHEKs) were isolated from normal adult human foreskin and cultured as described previously^21^. Experiments were conducted according to the principles of the Declaration of Helsinki and approved by the Ethics Committee of Mackay Memorial Hospital (13MMHIS048). Written informed consent was obtained from donors before experiments. NHEKs were cultured in Keratinocyte-SFM (Gibco BRL/Invitrogen, Carlsbad, CA, USA) supplemented with EGF (0.1–0.2 ng/ml), bovine pituitary extract (20–30 mg/ml), and 0.1% penicillin-streptomycin. In some experiments, culture medium without EGF was used. NHEKs were used for experiments within 3 passages. For cell differentiation, we used the confluence, PMA and calcium models as we previously described^16,22^.

### Reagents

Antibodies directed against JNK1, ERK2, PKCα, PKCδ, EGFR, TGase 1, and involucrin, and horseradish peroxidase-coupled secondary antibodies were purchased from Santa Cruz Biotechnology (Santa Cruz, CA, USA). Antibodies against human keratin 10 and loricrin were purchased from Covance (Emeryville, CA, USA). Antibodies against phosphorylated JNK, ERK, and EGFR were purchased from Cell Signaling Technology (Beverly, MA, USA). Enhanced chemiluminescence reagent was obtained from PerkinElmer (Wellesley, MA, USA). Myelin basic protein (MBP) was purchased from Sigma–Aldrich (St. Louis, MO, USA).

### Small interfering RNA (siRNA) transfection

siRNA experiments were performed by using siRNAs targeting human DcR3, PKCα, and PKCδ and a nontargeting siRNA as the control (Thermo Fisher Scientific, Lafayette, CO, USA). NHEKs at approximately 50% confluence were transfected with 100 nM siRNA with DharmaFECT Transfection Reagent following the manufacturer’s instructions. Cells were harvested at the indicated time points.

### Immunoblotting

Equal amounts of soluble protein were loaded and electrophoresed on SDS–PAGE gels and were then transferred to Immobilon-P membranes (Millipore, Billerica, MA, USA). Specific protein bands were detected with enhanced chemiluminescence detection reagent.

### Quantitative real-time RT–PCR analysis

The procedure was carried out as previously described, and the primers used to amplify the housekeeping gene cyclophilin A and human involucrin, TGase 1, keratin 10, loricrin, EGFR, amphiregulin, HB-EGF and transforming growth factor-α (TGF-α) were described previously^22,23^. The primers used to amplify DcR3 (NM_003823.3) were designed with Primer Express Software (Applied Biosystems, CA, USA).

### Crystal violet assay

To determine the relative cell number, NHEKs transfected with the indicated siRNA were cultured in medium with or without growth factors for 5 days. After rinsing with phosphate-buffered saline (PBS), cells were fixed with methanol for 10 min at room temperature and were then stained with 0.1% crystal violet. The relative cell number was determined by measuring the absorbance of the dissolved dye at 540 nm after elution with 33% acetic acid.

### Cell counting and cell cycle analysis

After DcR3 knockdown, NHEKs were cultured in medium with or without growth factors. To generate the cell growth curve, the number of cells per well was determined 2 and 5 days after knockdown, and the fold change relative to the initial number of cells was calculated. To determine the cell cycle distribution, cells were collected, washed with ice-cold PBS, and fixed with 70% (v/v) ethanol in PBS overnight at −20 °C. The supernatant was removed after centrifugation, and the pellet was resuspended in DNA extraction buffer (0.2 M Na_2_HPO_4_, 0.1 M citric acid (pH 7.8)). After another centrifugation step, the pellet was stained with 80 μg/ml PI, and the cell cycle distribution was analyzed by flow cytometry (BD, FACSCalibur) as described previously^16^.

### Enzyme-linked immunosorbent assay (ELISA)

Commercial ELISA kits (R&D Systems, Minneapolis, MN) were used to determine amphiregulin levels in cell culture supernatants according to the manufacturer’s instructions.

### PKC kinase activity assay

Equal amounts of soluble protein were used for measurement of total kinase activity with a PKC kinase activity kit (ENZO Life Sciences, Farmingdale, NY, USA) according to the manufacturer’s instructions. For the PKCα and PKCδ kinase assays, immunoprecipitation followed by an in vitro kinase assay was carried out as described previously^24^. An anti-PKC antibody was applied, and MBP was used as the substrate.

### Statistical analysis

Data are expressed as the mean ± S.E.M. of at least 3 independent experiments, and Student’s *t* test was used to assess the statistical significance of the differences. A *P* value < 0.05 was considered statistically significant.

## Results

### DcR3 mRNA and protein levels are decreased along with keratinocyte differentiation

**Fig. 1:**
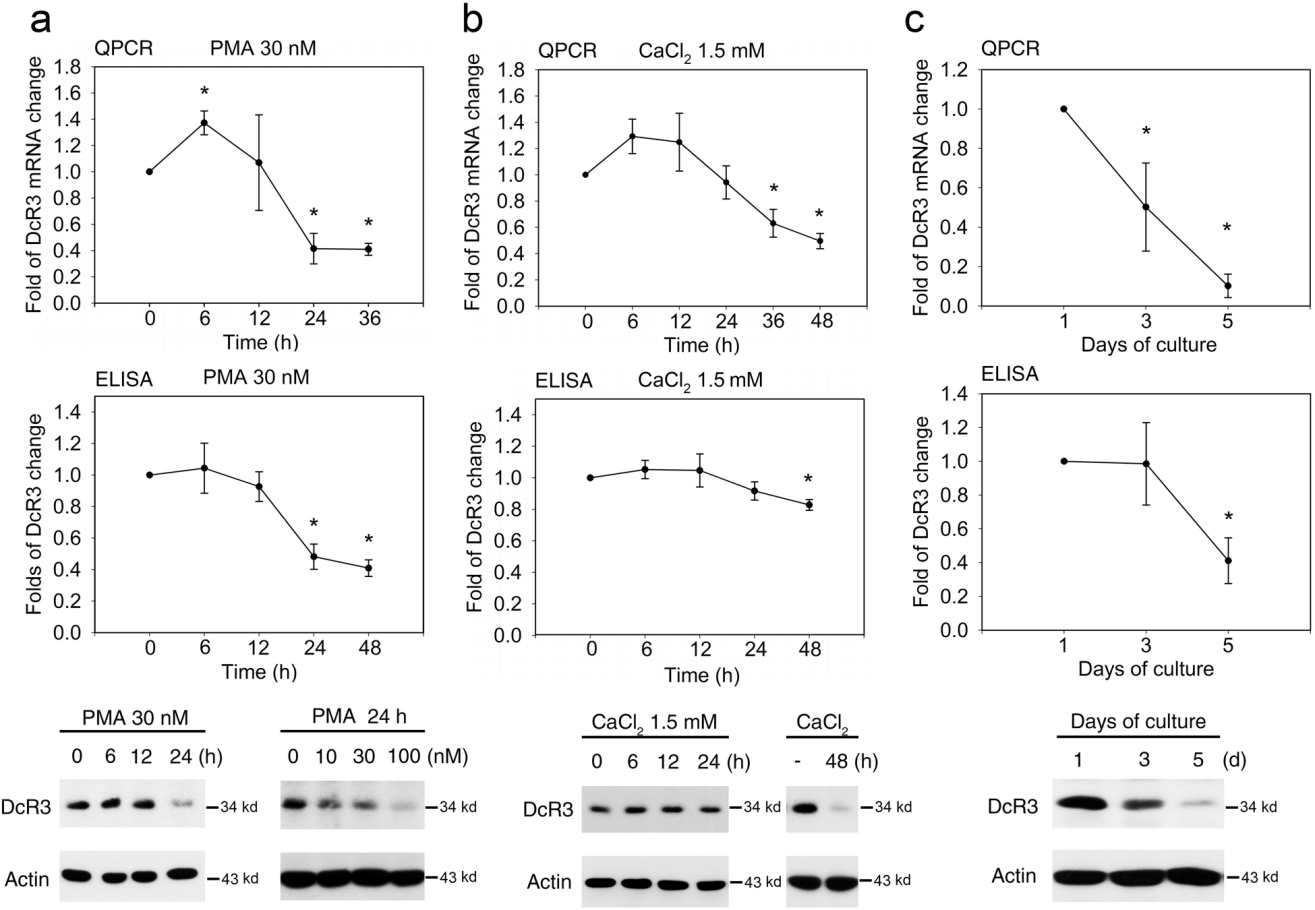
Decreased DcR3 expression in keratinocytes during terminal differentiation. NHEKs were differentiated by PMA treatment (30 nM) (**a)**, 1.5 mM CaCl_2_ treatment (**b**) or confluence (**c**). At different times, quantitative real-time RT–PCR was performed to evaluate DcR3 mRNA expression (upper panels), and ELISA (middle panels) and immunoblotting (lower panels) were used to determine the released soluble and intracellular DcR3 protein levels, respectively. *p < 0.05, indicating a significant change in DcR3 expression compared to the initial level.

To induce cell differentiation, three classical in vitro differentiation models—phorbol 12-myristate 13-acetate (PMA)-induced, calcium-induced and cell confluence-induced differentiation—were used. Our data showed that DcR3 mRNA expression was constitutively detected in proliferating NHEKs and that after PMA (30 nM) treatment, DcR3 mRNA expression was transiently increased within 6 h and was then time-dependently suppressed upon PMA-induced NHEKs differentiation (Fig. 1a, upper panel). Both the secreted and intracellular DcR3 protein levels, as measured by ELISA and immunoblotting (Fig. 1a, middle and lower panels), respectively, were reduced in a time-dependent manner after PMA treatment. In the calcium-induced differentiation model, we found that treatment with 1.5 mM extracellular calcium also resulted in transient upregulation of DcR3 mRNA expression at 6 h followed by a gradual decrease. A significant reduction in DcR3 mRNA expression was observed after 36 h of incubation (Fig. 1b, upper panel). Accordingly, significant reductions in DcR3 secretion (Fig. 1b, middle panel) and the intracellular DcR3 protein level (Fig. 1b, lower panel) were observed at 48 h. In comparing both models, we found that the onset of DcR3 downregulation induced by 1.5 mM calcium was later than that induced by PMA, i.e., 36–48 h vs. 24 h. Moreover, in the cell confluence-induced model established by plating NHEKs at a 4-fold higher density and culturing them for 5 days, the DcR3 mRNA (Fig. 1c, upper panel) and protein levels (Fig. 1c, middle and lower panels) were time-dependently decreased. Taken together, these findings indicate that the DcR3 gene and protein expression levels are attenuated when keratinocytes undergo differentiation.

### The reduction in EGFR expression accompanying keratinocyte terminal differentiation may contribute to downregulation of DcR3

**Fig. 2:**
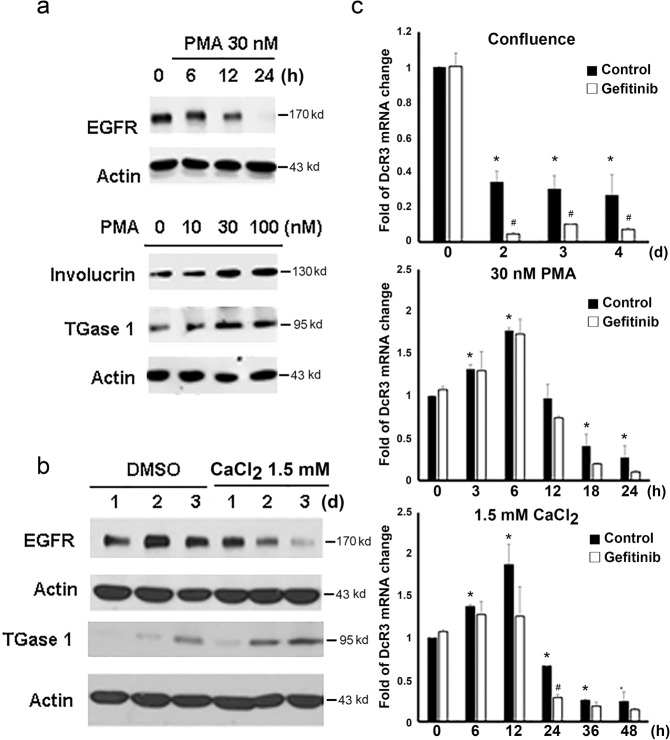
EGFR and DcR3 were simultaneously downregulated during keratinocyte differentiation. NHEKs were differentiated by PMA treatment (**a, c**), calcium treatment (**b, c**) or confluence (**c**) for different times. The protein levels of EGFR, involucrin and TGase 1 were determined by immunoblotting (**a, b**). In (**c**), differentiated keratinocytes were treated with gefitinib (10 µM), and the DcR3 mRNA level was determined by real-time PCR. *p < 0.05, indicating a significant change in DcR3 expression compared to the initial level. #*p* < 0.05, indicating that gefitinib significantly further decreased the DcR3 mRNA level during differentiation.

In our previous study, we demonstrated that DcR3 gene transcription in keratinocytes is induced by EGFR ligands (e.g., EGF and TGFα) and is mediated by NF-κB and JNK signaling^21^. Because we found that EGFR protein expression is also downregulated along with confluence-induced keratinocyte differentiation and that this event is accompanied by enhanced expression of the differentiation markers K10 and loricrin^16^, we sought to understand the role of DcR3 in keratinocyte differentiation and its relationship to EGFR. First, we determined the changes in EGFR expression in the PMA- and calcium-induced differentiation models. We found that similar to the effect observed above in the confluence-induced model, EGFR protein expression was time-dependently decreased by treatment with 30 nM PMA, accompanied by increased involucrin and TGase 1 expression, during cell differentiation (Fig. 2a). Likewise, 1.5 mM calcium treatment time-dependently downregulated EGFR but upregulated TGase 1 (Fig. 2b).

Next, we treated NHEKs with the EGFR inhibitor gefitinib to determine whether endogenous EGF/EGFR autocrine activity is involved in regulating DcR3 expression, as we previously observed for exogenous EGF in keratinocytes^21^. We found that gefitinib enhanced confluence-induced and calcium-induced but not PMA-induced suppression of DcR3 mRNA expression **(**Fig. 2c**)**. Moreover, the transient effects of PMA (within 6 h) and calcium (within 12 h) on upregulating DcR3 gene expression were not altered by gefitinib. Collectively, these data suggest that the decrease in DcR3 expression after progressively longer periods under each differentiation condition might be ascribed to decreased EGFR activation, particularly in the confluence-induced and calcium-induced models. On the other hand, the transient induction of DcR3 expression by PMA (within 6 h) and calcium (within 12 h) was independent of EGFR activity.

### DcR3 expression in keratinocytes modulates the expression of differentiation markers

**Fig. 3:**
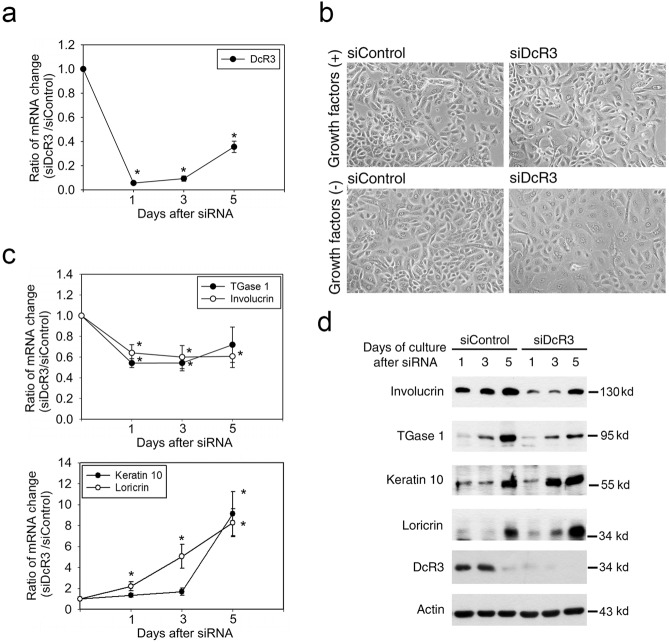
DcR3 differentially regulated the expression of differentiation markers in epidermal keratinocytes. After knocking down DcR3, quantitative real-time RT–PCR was performed to detect the mRNA expression of DcR3 (**a**) and differentiation markers, including TGase 1, involucrin, keratin 10 and loricrin (**c**). The ratio of mRNA expression in NHEKs transfected with DcR3 siRNA to that in NHEKs transfected with control siRNA (siDcR3/siControl) was determined. *p < 0.05 when the ratio was significantly higher or lower than 1. **b** NHEKs treated with DcR3 siRNA or control siRNA were cultured in medium with or without growth factors. Cell morphology was examined by microscopy. **d** After knocking down DcR3, protein expression was determined by immunoblotting.

Due to the abundant expression of DcR3 in NHEKs, we knocked down DcR3 expression by using DcR3 siRNA to investigate the role of DcR3 in keratinocyte differentiation by measuring the mRNA expression of the typical differentiation markers keratin 10, involucrin, loricrin and TGase 1. As shown in Fig. 3a, efficient knockdown of DcR3 mRNA expression was confirmed by quantitative real-time PCR. We found that compared to that of control cells, the cytoplasm of DcR3-silenced cells was more abundant under culture in starvation medium (Fig. 3b). During the growth of cells to confluence, the mRNA expression levels of involucrin and TGase 1 were reduced but those of keratin 10 and loricrin were increased in the DcR3-silenced group compared to the control group **(**Fig. 3c**)**. The immunoblotting results further revealed correlated changes in the protein expression levels of these differentiation markers during growth to confluence. The confluence-dependent increases in involucrin and TGase 1 expression were reduced but the increases in keratin 10 and loricrin expression were enhanced by siDcR3 treatment (Fig. 3d). These data suggest that DcR3 can differentially regulate the expression of differentiation markers. Because DcR3 expression is controlled by EGFR ligands^21^ and EGFR signaling is crucial for the differential regulation of keratinocyte differentiation molecules, i.e., upregulation of involucrin and TGase but downregulation of keratin 10 and loricrin^16,25–27^, we suggest an association between DcR3 and EGFR in the regulation of keratinocyte differentiation.

### DcR3 reciprocally and positively regulates EGFR signaling

**Fig. 4:**
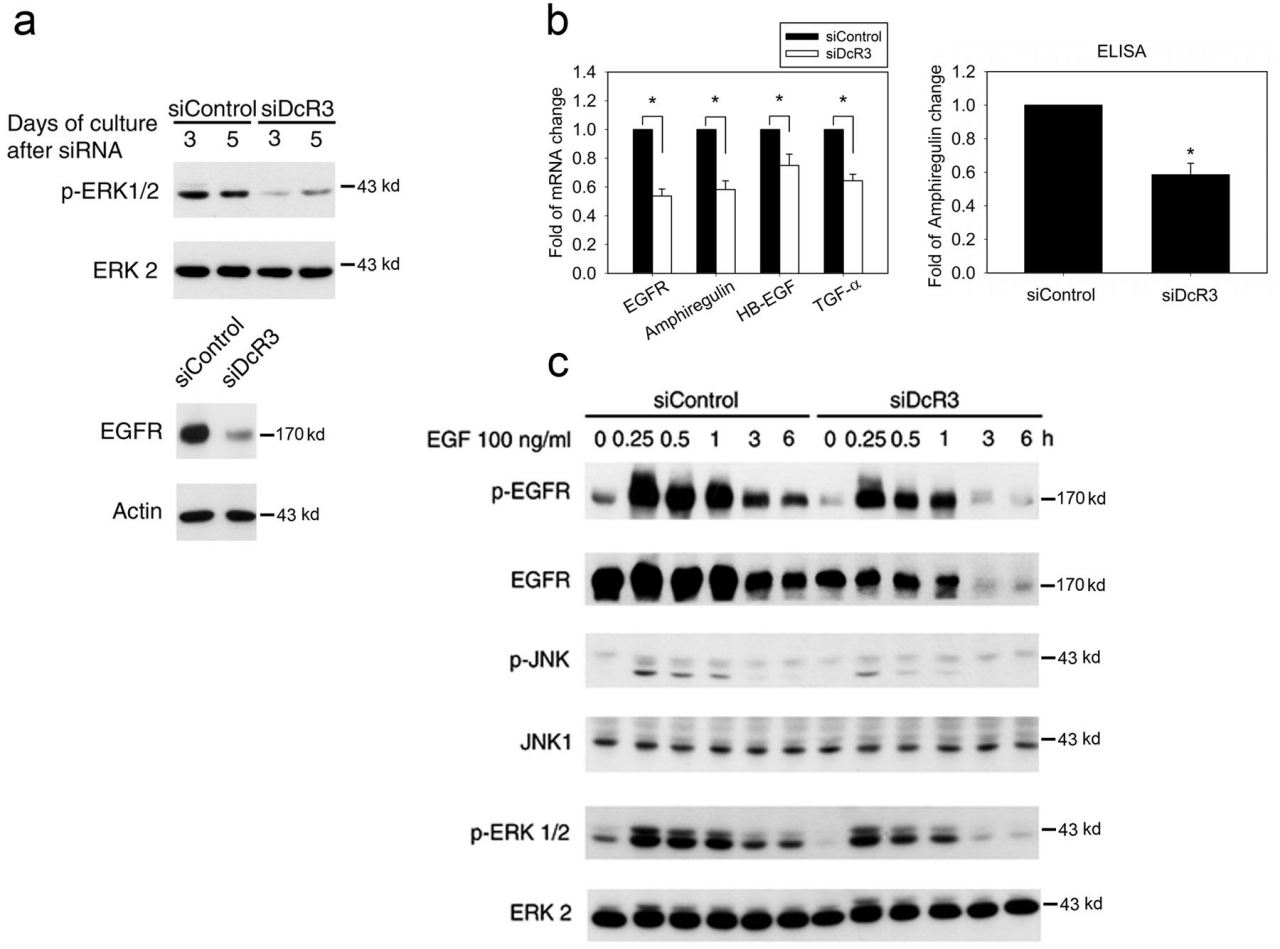
DcR3 positively regulated EGFR ligand expression and EGFR signaling. After DcR3 silencing, NHEKs were harvested for immunoblotting (**a**) and real-time PCR (**b**, left panel), and the amphiregulin level in the culture medium was measured by ELISA (**b**, right panel). **c** After one day of transfection with siControl or siDcR3, NHEKs were starved for 48 h and were then treated with EGF (100 ng/ml) for different times. Activation of EGFR, ERK and JNK signaling was determined by immunoblotting. *p < 0.05, indicating significant decreases in EGFR and EGFR ligand expression and amphiregulin secretion in DcR3-silenced cells compared to control cells.

After identifying the link between DcR3 and EGFR, we asked whether DcR3 might exert a feedback effect on EGFR. To this end, we evaluated the effects of DcR3 silencing on EGFR activation induced by endogenous ligands or exogenous EGF treatment. We found that DcR3 silencing reduced EGFR protein expression and constitutive ERK activation (Fig. 4a). Moreover, the mRNA expression of EGFR and its ligands (amphiregulin, HB-EGF and TGF-α) was attenuated in cells with DcR3 silencing. In addition, the extracellular amphiregulin protein level was reduced (Fig. 4b). Moreover, consistent with these findings, exogenous EGF-induced EGFR phosphorylation and downstream ERK and JNK signaling were attenuated in DcR3-silenced cells (Fig. 4c). These findings suggest the existence of a positive feedback loop between EGFR signaling and DcR3 expression in keratinocytes.

### DcR3 does not affect keratinocyte proliferation

**Fig. 5:**
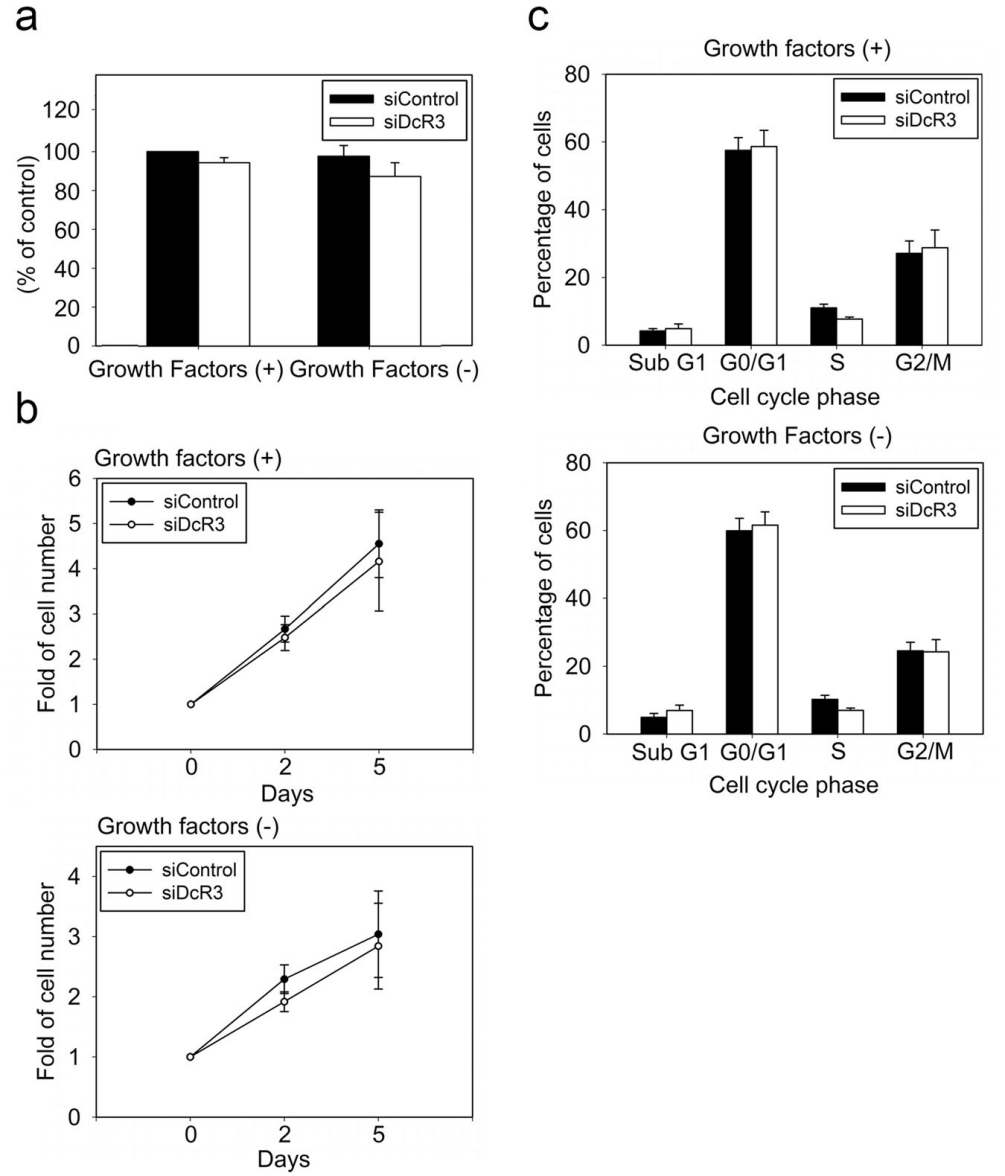
DcR3 silencing did not affect keratinocyte growth. NHEKs transfected with siControl or siDcR3 were cultured in medium with or without growth factors. Five days later, a crystal violet assay was performed (**a**), and the cell cycle distribution was analyzed by flow cytometry (**c**). **b** Cell growth curves were generated by cell counting and are presented as the fold increase in the cell number compared with the initial cell number before siRNA transfection.

Generally, the differentiation process in most cell types is negatively controlled by cell growth. Although keratinocyte differentiation is different from the conventional scenario and is potentiated by cell confluence, we sought to understand whether DcR3 might regulate keratinocyte proliferation. Via a crystal violet assay, we found that when NHEKs were incubated in culture medium either with or without growth factors for 5 days, DcR3 silencing did not induce a significant change in cell viability (Fig. 5a). In addition, the proliferation of keratinocytes, as evaluated by direct cell counting, was not changed by DcR3 silencing upon culture in either the absence or presence of growth factors for 5 days (Fig. 5b). Likewise, data showing the cell populations in different cell cycle phases revealed no differences between control and DcR3-silenced cells (Fig. 5c). Collectively, these findings suggest that DcR3-mediated regulation of differentiation marker expression is not linked to cell growth.

### DcR3 regulation of keratinocyte differentiation might occur via intracellular and extracellular actions

**Fig. 6:**
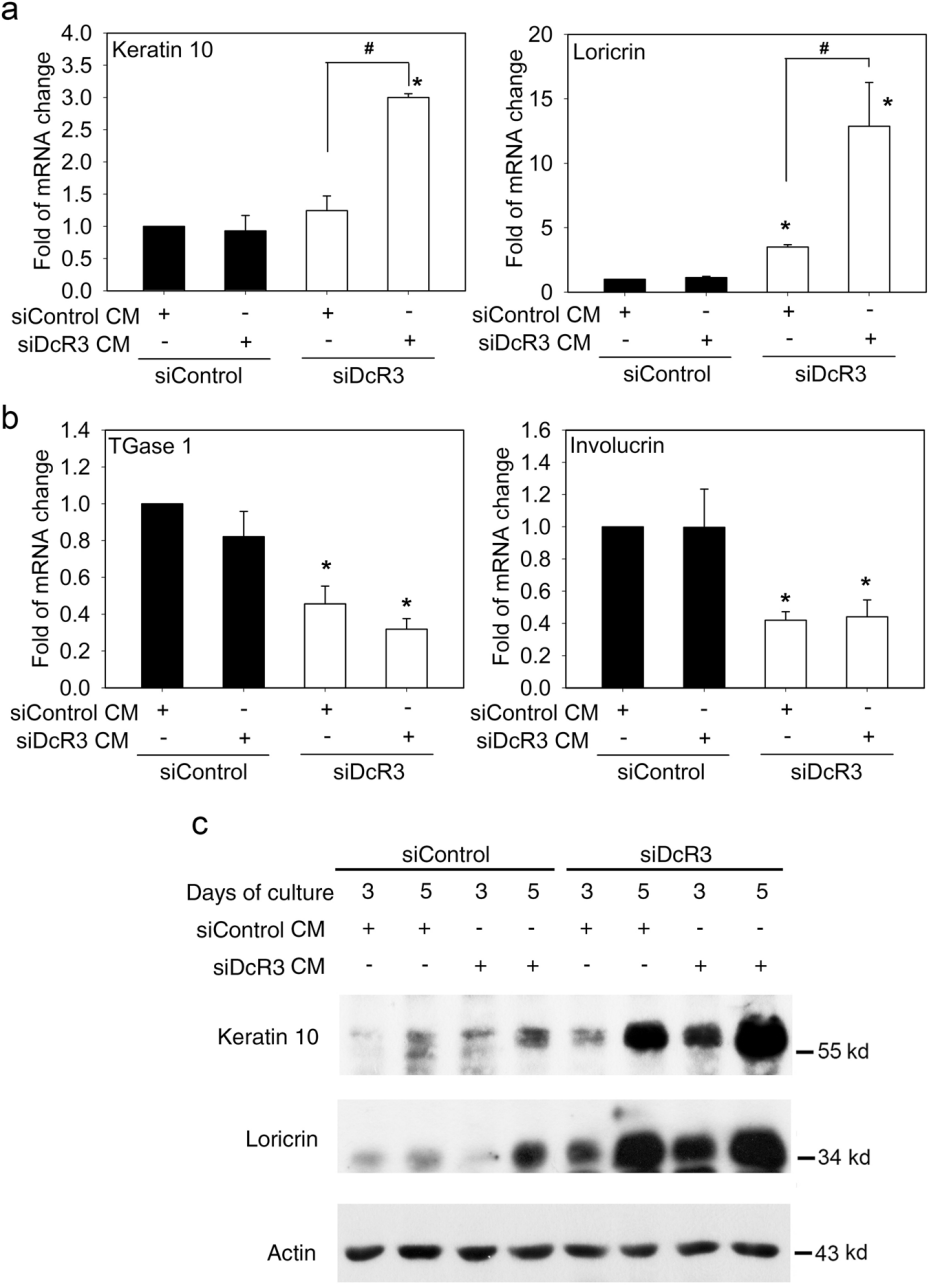
Extracellular DcR3 reversed the effect of DcR3 silencing on keratinocyte differentiation in a gene-specific manner. One day after siRNA transfection, NHEKs transfected with control siRNA (siControl) or DcR3 siRNA (siDcR3) were treated with conditioned medium (CM) from NHEKs transfected with control siRNA (siControl CM) or with DcR3 siRNA (siDcR3 CM). **a, b** The procedure was repeated daily until the 4th day after transfection, when mRNA was isolated for real-time PCR analysis. *p < 0.05, indicating a significant change in gene expression by DcR3 silencing. ^#^*p* < 0.05, indicating the reversal effect of siControl CM. **c** Cells were collected on the 3rd day and 5th day after siRNA transfection, and immunoblotting was then performed.

DcR3 is a soluble factor, and extracellular DcR3 has been shown to produce biological responses via the neutralization of identified TNF superfamily ligands, i.e., FasL, LIGHT and TL1A, or via a nondecoy function^4^. To understand if DcR3’s action is mediated via an extracellular site, we first collected the conditioned medium (CM) of NHEKs with or without DcR3 silencing (i.e., siDcR3 CM, containing barely any DcR3; and siControl CM, containing a high amount of DcR3, respectively). Then, we treated siDcR3 cells separately with each type of CM and tested the ability of these CMs to restore the actions of DcR3 in siDcR3 cells. As shown in Fig. 6a, we found that the increases in the keratin 10 and loricrin mRNA levels caused by DcR3 silencing were inhibited in cells cultured with siControl CM. In contrast, siControl CM could not reverse the downregulation of TGase 1 and involucrin (Fig. 6b). The immunoblot results confirmed the reversal of the changes in the expression of the differentiation markers keratin 10 and loricrin by siControl CM (Fig. 6c). Based on these findings, we suggest that DcR3 can regulate keratin 10 and loricrin gene expression via an extracellular mechanism, while its regulation of TGase 1 and involucrin gene expression might occur via an intracellular mechanism.

### DcR3 regulates keratinocyte differentiation via modulation of PKC activity

**Fig. 7:**
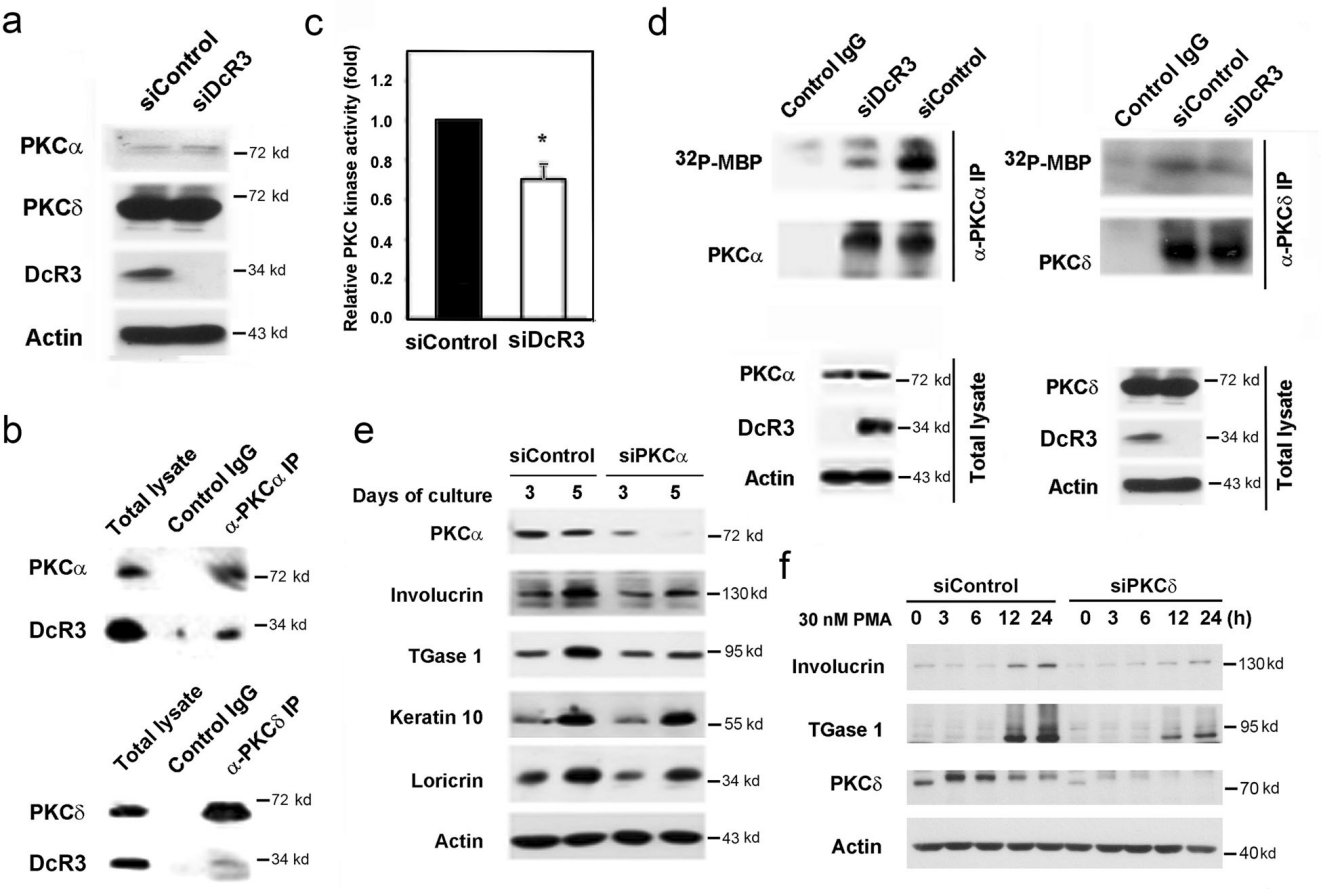
DcR3 interacted with PKCα and PKCδ and modulated kinase activity. Three days after siRNA transfection, NHEKs were harvested for determination of PKCα and PKCδ protein expression by immunoblotting (**a**) and determination of the basal kinase activity of total PKC by a commercial PKC kinase activity kit (**c**). *p < 0.05, indicating a significant reduction in PKC activity in cells with DcR3 silencing compared with control cells. **b** PKCα and PKCδ were immunoprecipitated, and immunoblotting with an anti-DcR3 antibody was then performed to detect the interaction. **d** Three days after DcR3 knockdown, PKCα and PKCδ were individually immunoprecipitated and subjected to an in vitro kinase assay. **e** NHEKs w**e**re harvested on the 3rd day and 5th day after PKCα knockdown by siRNA transfection. Immunoblotting was performed to evaluate protein expression in NHEKs transfected with control siRNA (siControl) and PKC siRNA. **f** After PKCδ knockdown, NHEKs were treated with 30 nM PMA and harvested at the indicated time points. Immunoblotting was performed to evaluate protein expression.

To understand the possible molecular mechanism by which intracellular DcR3 controls gene expression, we considered the potential actions of PKC isoforms. Previously, PKCα and PKCδ have been documented to control keratinocyte differentiation^28–30^, and our previous study revealed the ability of exogenous DcR3 to activate PKC in monocytes^31^. Therefore, we clarified the functional relationship between DcR3 and PKC in keratinocyte differentiation. We first determined the effect of DcR3 silencing on PKC isoform expression. As shown in Fig. 7a, the protein levels of PKCα and PKCδ were not changed by DcR3 silencing. Of note, we found that DcR3 is associated with both the α and δ isoforms of PKC (Fig. 7b). We asked whether DcR3 might affect PKC activity via a protein–protein interaction. Thus, we conducted an in vitro kinase assay and found that the total PKC activity in siDcR3 cells was reduced compared to that in the control group (Fig. 7c). We further conducted immunoprecipitation of PKCα and PKCδ for an in vitro kinase assay and, consistent with the above results, found that the activity of both PKCα and PKCδ was decreased in siDcR3 cells (Fig. 7d). To verify the roles of these PKC isoforms in regulating the above-tested genes, we knocked down PKCα and PKCδ. siPKCα reduced confluence-induced involucrin and TGase 1 protein expression but affected keratin 10 and loricrin expression to a lesser degree (Fig. 7e). On the other hand, siPKCδ was shown to reduce PMA-induced involucrin and TGase 1 expression (Fig. 7f).

## Discussion

DcR3 has been demonstrated to perform multiple immune system-related functions upon inflammation and is a critical factor involved in the pathogenesis of multiple diseases in addition to cancers. It is regarded as a potential biomarker for predicting inflammatory disease progression and cancer metastasis^3^. Although DcR3 has a decoy function in neutralizing FasL, LIGHT and TL1A, recent evidence indicates that DcR3 can also act as an extracellular effector of “nondecoy” biological functions^32–34^. A further study using a fragment of the DcR3 protein without a decoy function indicated that the DcR3-mediated nondecoy function is mediated via crosslinking of heparin sulfate proteoglycans such as syndecans and CD44v3^4^. In addition to acting as an inflammatory biomarker, DcR3 serves as a novel physiological regulator in keratinocytes. To date, the study of DcR3 in skin is still quite limited. Previously, we and others detected DcR3 in human primary keratinocytes and found that its expression was decreased by UV light^18^ but increased by EGF and TGF-α^21^. To date, the cellular function of DcR3 in keratinocytes remains unclear. In this study, quantitative real-time PCR analysis revealed downregulation of DcR3 mRNA expression during keratinocyte differentiation induced by calcium, PMA, and cell confluence. The immunoblot results further revealed decreased DcR3 protein expression in keratinocytes during differentiation. We also found, for the first time, novel intracellular actions of DcR3 in modulating the gene expression of keratinocyte markers, suggesting that DcR3 can regulate cellular functions in addition to performing decoy and nondecoy functions as a secreted protein. In keratinocytes, intracellular DcR3 can enhance PKCα/δ activity, which might be involved in controlling keratinocyte homeostasis during differentiation.

In normal skin, EGFR activity is usually high in the basal layer but low in the upper layers. In psoriasis, maintenance of EGFR activity can be observed in the upper epidermal layer^35–37^. Our previous study showed that DcR3 is mainly expressed in the upper epidermal layer in psoriatic skin^21^, which is characterized by reduced loricrin and keratin 10 expression but increased TGase 1 and involucrin expression^38–40^. In this study, we demonstrated that DcR3, like EGF, as previously reported, is involved in upregulating TGase 1 and involucrin but downregulating keratin10 and loricrin gene expression. The alterations in these differentiation genes upon DcR3 silencing actually mimic the effects of EGFR inhibition. In this context, previous studies demonstrated that EGFR activity is involved in the differential regulation of keratinocyte differentiation genes, i.e., upregulation of involucrin and TGase 1 but downregulation of keratin 10 and loricrin^16,25–27,41^. Moreover, our data revealed that DcR3 is involved in maintaining constitutive EGFR activity, possibly by promoting the expression of endogenous EGFR ligands (amphiregulin, HB-EGF and TGF-α) and EGFR. The reduced EGFR expression caused by DcR3 silencing also led to the attenuation of EGFR signaling elicited by EGF. Thus, we suggest that the positive regulatory role of DcR3 in EGFR signaling might involve mediation of EGFR-dependent regulation of keratinocyte differentiation. On the other hand, upregulation of DcR3 expression by EGF and TGF-α via EGFR-mediated IKK/NF-κB and JNK pathways has been demonstrated in keratinocytes^21^. Therefore, we suggest that DcR3 and EGFR activation form a positive feedback loop and that DcR3 might mediate the regulatory functions of EGFR in keratinocyte differentiation, which might accelerate the pathogenesis of skin disorders such as psoriasis. Accordingly, in addition to the previous idea that combined treatment with a differentiation-promoting agent and an EGFR inhibitor may be an additional approach for the management of hyperproliferative skin diseases^41^, silencing DcR3 provides another promising alternative strategy.

In this study, we also excluded the possibility that DcR3-mediated regulation of differentiation marker expression results from an indirect action on cell growth. We found that DcR3 silencing does not alter cell growth, possibly because the initiation of keratinocyte differentiation is not inseparably associated with cell cycle arrest^42^. On the other hand, the positive EGFR-DcR3 loop mentioned above was verified by additional findings. First, during keratinocyte differentiation induced by PMA treatment, calcium treatment or cell confluence, EGFR activity was time-dependently decreased gradually. This change in EGFR activity kinetics occurred in parallel to the downregulation of DcR3 mRNA expression. Second, the observation that suppression of DcR3 gene expression was enhanced by the EGFR inhibitor gefitinib during differentiation in the models suggests the role of EGFR in DcR3 gene expression. However, before the time-dependent decrease in DcR3 during keratinocyte differentiation, we observed transient induction of DcR3 expression by PMA (within 6 h) and calcium (within 12 h) treatment, and this effect was independent of EGFR activity. Currently, we do not have other data to clarify the mechanisms and cellular functions of this transient increase in DcR3 gene expression.

To date, the nondecoy functions of DcR3 responsible for some cellular functions and therapeutic potentials have been documented. These include driving dendritic cell and macrophage polarization toward Th2 phenotypes and M2-like phenotypes, respectively^32,34^. Despite accumulating lines of evidence indicating the effector function of extracellular DcR3^3^, only a limited understanding of the mechanism of action has been obtained. In this respect, extracellular DcR3 can interact with heparin sulfate proteoglycans (HSPGs), such as syndecans and CD44v3, in myeloid cells^4^, induce M2 macrophage polarization via epigenetic regulation^33,34^, and induce monocyte adhesion via activation of FAK^31^. Despite these findings, the possible intracellular action of DcR3 is totally unknown. In this study, we identified a novel intracellular target of DcR3. We demonstrated the interactions of intracellular DcR3 with PKCα and PKCδ and found that these interactions contribute to constitutive PKC activation. Because resting PKC activity can be controlled by EGFR^43^ and regulate keratinocyte differentiation^44,45^, we speculate that DcR3-mediated regulation of keratinocyte differentiation might involve PKC activity.

Our study also showed the role of the extracellular action of DcR3 in keratinocyte differentiation. Via the conditioned medium approach, we found that exogenous DcR3 reversed the effects of DcR3 silencing on upregulating keratin 10 and loricrin gene expression but failed to change DcR3 silencing-induced downregulation of TGase 1 and involucrin. These findings not only reveal the gene-specific regulation by DcR3 and EGF but also indicate that DcR3 serves as an effector via its extracellular action. Several lines of evidence indicate that TL1A may be involved in the pathogenesis of psoriasis^20,46^. Other ligands neutralized by DcR3, such as FasL and LIGHT, have been shown to regulate keratinocyte death^46^, keratinocyte-mediated skin inflammation and fibrosis^47,48^, but their effects on keratinocyte differentiation are unknown. Although we cannot exclude the possibility of interactions between DcR3 and these ligands in regulating keratinocyte differentiation, more studies are required to explore other possible soluble factors regulated by DcR3 in keratinocytes.

In summary, we demonstrated a new function of DcR3 in impairing keratinocyte differentiation. Via a reciprocal and positive feedback loop formed with EGFR, DcR3 promotes TGase 1 and involucrin expression, and it can also interact with the PKCα/δ isoforms to support PKCα/δ activity. In addition, extracellular DcR3 can negatively regulate keratin 10 and loricrin expression via an unidentified mechanism of action. Collectively, these findings indicate that keratinocyte-derived DcR3 can integrate EGFR and PKC signaling to promote TGase 1 and involucrin expression but inhibit keratin 10 and loricrin expression via extracellular and intracellular mechanisms of action. Dysregulation of the EGFR-DcR3 axis leads to impaired keratinocyte differentiation in psoriasis.
